# Is the Occurrence of the Sticking Region in Maximum Smith Machine Squats the Result of Diminishing Potentiation and Co-Contraction of the Prime Movers among Recreationally Resistance Trained Males?

**DOI:** 10.3390/ijerph18031366

**Published:** 2021-02-02

**Authors:** Roland van den Tillaar, Eirik Lindset Kristiansen, Stian Larsen

**Affiliations:** Department of Sport Sciences and Physical Education, Nord University, 7600 Levanger, Norway; ek1105@hotmail.com (E.L.K.); stianandrelarsen@live.no (S.L.)

**Keywords:** electromyography (EMG), isometric force, strength

## Abstract

This study compared the kinetics, barbell, and joint kinematics and muscle activation patterns between a one-repetition maximum (1-RM) Smith machine squat and isometric squats performed at 10 different heights from the lowest barbell height. The aim was to investigate if force output is lowest in the sticking region, indicating that this is a poor biomechanical region. Twelve resistance trained males (age: 22 ± 5 years, mass: 83.5 ± 39 kg, height: 1.81 ± 0.20 m) were tested. A repeated two-way analysis of variance showed that Force output decreased in the sticking region for the 1-RM trial, while for the isometric trials, force output was lowest between 0–15 cm from the lowest barbell height, data that support the sticking region is a poor biomechanical region. Almost all muscles showed higher activity at 1-RM compared with isometric attempts (*p* < 0.05). The quadriceps activity decreased, and the gluteus maximus and shank muscle activity increased with increasing height (*p* ≤ 0.024). Moreover, the vastus muscles decreased only for the 1-RM trial while remaining stable at the same positions in the isometric trials (*p* = 0.04), indicating that potentiation occurs. Our findings suggest that a co-contraction between the hip and knee extensors, together with potentiation from the vastus muscles during ascent, creates a poor biomechanical region for force output, and thereby the sticking region among recreationally resistance trained males during 1-RM Smith machine squats.

## 1. Introduction

Different variations of the squat are popular to enhance maximal strength in the lower extremities during resistance training [1]. A typical two-legged back squat is performed when the barbell is lowered by flexing the knees and hips during descent and thereafter extending the joint during ascent so that the joint is fully extended. At maximal and submaximal attempts, a sticking region has been shown to occur during ascent [2,3]. The sticking region is considered the weakest link during ascent and it is often there where the lift fails [4,5,6]. It was hypothesised that during the sticking region, the involved muscles could have a disadvantageous length to produce force, creating a poor biomechanical region to exert force in the bench press exercise [2,3]. In addition, it was proposed that the start of the sticking region occurs because the enhanced force during ascent (potentiation) is diminished [4]. To test this hypothesis, van den Tillaar et al. [5] examined a regular one-repetition maximum (1-RM) free weight bench press and compared it to isometric bench presses performed at 12 different positions, covering the full range of motion during ascent. They found a region of lower force output during the sticking region; additionally, the activity of all four muscles showed the same pattern in the isometric and 1-RM attempts. Therefore, they concluded that the sticking region was most likely the result of a poor mechanical force position, but diminishing potentiation effects could not explain the existence of the sticking region in the bench press exercise.

In the squat exercise, other muscles are responsible for overcoming the ascent and completing the lift. Van den Tillaar [7] investigated muscle activity in squats during the sticking region and found that the quadriceps and plantar flexors had increased activity, while the gluteus maximus had decreased activity during ascent. Therefore, he proposed that the timing in co-contraction between the hip and knee extensors, together with large moment arms, could be the reason for the occurrence of sticking region in squats. Similar studies have also reported the same development in muscle activity between the hip and knee extensors [8,9,10,11]. However, the aforementioned studies did not investigate the force output in and around the sticking region. Furthermore, these studies were performed with both a descent and ascent movement, which can cause diminishing potentiation. Because such a mechanism cannot play a significant role during isometric contractions, investigating isometric contractions at various positions during the squat exercise could be used to investigate whether the sticking region is caused by potentiation, or if the occurrence of the sticking region is due to only a poor mechanical force position in the squat exercise. It could reveal if force output decreases at specific joint angles, as suggested by van den Tillaar [7], a phenomenon that causes longer moment arms for muscles and, thereby, the potential for a lower force output. Previous studies [7,8,10,12] have revealed muscle activity for each region, but not around the different angles. Information from the present study could help coaches and athletes understand if the sticking region occurs at specific joint angles and if force output is lower in the sticking region. Potentially, this information could help them to target specific muscles and angles to enhance squat performance during training and thereby increase lower limb strength in general.

We aimed to compare the kinetics, barbell kinematics, joint kinematics, and muscle activation patterns of maximal Smith machine squats to several isometric squats performed at 10 different positions from the lowest barbell height, covering the full range of motion during ascent. We hypothesised that the force output is the lowest at heights of the sticking region in the isometric contractions, which supports the theory that the sticking region is due to a poor mechanical position as shown in earlier findings on the bench press [5]. Furthermore, we expected differences in force output and muscle activation between maximal squats and isometric squats due to potentiation and increased muscle activation due to the descending phase in maximal squats [13].

## 2. Materials and Methods

The compare the kinetics, barbell kinematics, joint kinematics, and muscle activation patterns of maximal Smith machine squats to several isometric squats performed a within subject design was used in which each subject tested their 1-RM which was compared with the different isometric squat performance in one test session.

Twelve recreationally resistance-trained males (age: 22 ± 5 years, mass: 83.5 ± 39 kg, height: 1.81 ± 0.20 m), with at least 3 years of strength training history, participated in this study. The participants did not perform any resistance training exercises targeting the lower extremities in the 48 h before the testing session. The participants had no history of surgery or pain in the spine and lower extremities. Informed consent was obtained from all participant involved in the study, which was approved by the Norwegian Center for Research Data project number: 991974 and performed according to the Declaration of Helsinki of 2013.

Before the test session, the participants participated in a familiarisation session to establish their 1-RM using the Smith machine (Powerline Smith Machine model: PSM144X, Body-Solid, Forest Park, IL, USA). Before each session, they performed a standardised warm-up, followed by the assumed 1-RM (based on their previous experience and familiarisation test) in squats. The load was increased or decreased by 2.5 or 5 kg until the actual 1-RM was obtained (1–3 attempts) with 5 min rest between each attempt to avoid fatigue and obtain maximal 1-RM performance [14]. The participants stood on a force plate (Ergotest Innovation AS, Stathelle, Norway) sampling at 1000 Hz, with their preferred stance width in which they felt comfortable to perform the 1-RM lifts. This position was then controlled and used for all the isometric attempts. During the 1-RM attempts, the minimum depth requirement was that the knee joint was visually lower than 90 degrees and, if possible, the hip joint was visually lower than the knee joint according to the International Powerlifting Federation (IPF). The depth was measured and marked with a horizontal rubber band, which the participants had to touch with the proximal part of their hamstring before starting the ascending movement during 1-RM testing. Ten minutes after 1-RM testing, maximal isometric force output at every 5 cm from the lowest barbell height during the 1-RM to the fully upright position was tested in the Smith machine in random order. The Smith machine barbell was locked at each 5 cm height to simulate the full range of motion during the upward movement of the squat and the participant performed an isometric squat for 3 s with the same procedure as described by van den Tillaar, Saeterbakken and Ettema [5] for a bench press exercise. The pause between the different distances was approximately 5 min to avoid fatigue.

Before the 1-RM experimental test, the skin was prepared (shaved, washed with alcohol, and abraded) for placement of gel-coated surface electromyography (EMG) electrodes. The electrodes were placed on the right side of the body. The electrode pads (11 mm contact diameter, 20 mm centre-to-centre distance) were placed in the presumed direction of the underlying muscle fibres with a centre-to-centre distance of 2.0 cm. Self-adhesive electrodes (Dri-Stick Silver circular sEMG Electrodes AE-131, NeuroDyne Medical, Cambridge, MA, USA) were positioned on the belly of nine muscles: (a) vastus medialis, (b) vastus lateralis, (c) rectus femoris, (d) lateral side of the gastrocnemius, (e) gluteus maximus, (f) semitendinosus, (g) the long head of the biceps femoris, (h) soleus, and (i) erector spinae. Electrodes were placed according to the recommendations of SENIAM [15]. To minimise noise induced from external sources, the EMG raw signal was amplified and filtered using a preamplifier located as close to the pickup point as possible. The common-mode rejection ratio (CMRR) was 106 dB and the input impedance between each electrode pair was >10^12^ Ω. The EMG signals were sampled at a rate of 1000 Hz with Musclelab v.10.190 (Ergotest Innovation AS, Stathelle, Norway). Signals were band-pass filtered (fourth-order Butterworth filter) with a cut off frequency of 20 and 500 Hz, rectified, integrated, and converted to the root mean-square (RMS) signals using a hardware circuit network (frequency response 450 kHz, averaging constant 12 ms, total error ± 0.5%) [16].

To locate possible differences in muscle activity and force output during the 1-RM squat movement, we calculated the average force and RMS of each muscle for a 2-cm movement range around the same distances as in the isometric conditions. From the isometric conditions, we used the highest force output over approximately 1 s, together with the RMS muscle activity during this time, and compared it with the equivalent 1-RM height. We normalised the RMS muscle activity by using the highest RMS activity during one of the isometric conditions for each muscle. During the 1-RM and isometric attempts, the maximal force output was measured with a strain gauge force plate (Ergotest Innovation AS, Stathelle, Norway), placed directly under the feet in line with the barbell of the Smith machine at a 1000 Hz sampling rate.

**Figure 1:**
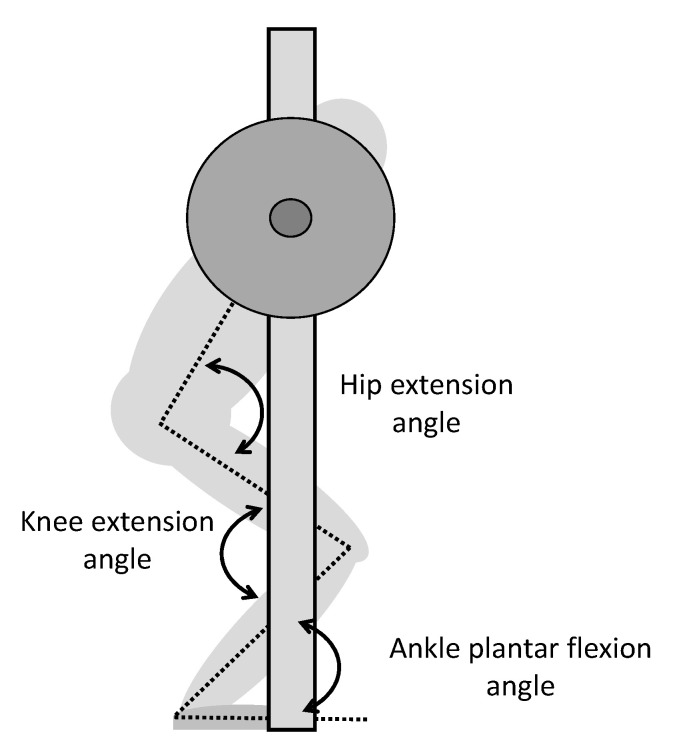
Definition of hip extension, knee extension, and ankle plantar flexion during squats in a Smith machine.

To measure the joint kinematics during the 1-RM and isometric conditions, we used a three-dimensional (3D) motion capture system (Qualysis, Gothenburg, Sweden), with eight cameras sampling at a frequency of 500 Hz, to track reflective markers, creating a 3D positional measurement. The markers were placed, one on each side of the body, on the lateral tip of the acromion, the iliac crest, the greater trochanter, the lateral and medial condyle of the knee, the lateral and medial malleolus, and the distal ends of the first and fifth metatarsals. Two markers were also placed on the middle of the barbell between the hands and shoulders 80 cm apart, to track barbell displacement. Segments of the feet, lower and upper leg, pelvis, and trunk were made in the Visual 3D v5 software (C-Motion, Germantown, MD, USA). We calculated barbell position and velocity and joint angles of hip extension, knee extension, and plantar flexion using the Visual 3D software. Joint angles were estimates of the anatomical angles calculated from lines formed between the centre of reflective markers. We recorded the joint angles of hip extension, knee extension, and ankle plantar flexion (Figure 1) at each isometric condition and used them for further analysis. During 1-RM testing, we measured joint kinematics at the following events: the lowest barbell point (v_0_), the first peak barbell velocity (v_max1_), the first local minimal barbell velocity (v_min_), and the second maximal barbell peak velocity (v_max2_). These events identify the start and end of reps and the pre-sticking, sticking, and post-sticking regions [8]. We synchronised the 3D motion capture system with the force plate and EMG recordings, using the Musclelab 6000 system (Ergotest Innovation AS, Stathelle, Norway).

### Data Analysis

We assessed normality using the Shapiro–Wilk test of normality. To assess differences in force output and muscle activity between the 1-RM at the different distances and the isometric attempts, we performed a repeated design two-way (condition: 1-RM-isometric) × 10 (distance: 0–45 cm) analysis of variance (ANOVA) for each of the eight muscles. We used a one-way ANOVA to compare the joint angles at the different isometric contraction heights. We conducted Holm–Bonferroni post hoc analyses to determine pairwise differences. All results are presented as the mean ± standard deviation (SD). If the sphericity assumption was violated, the Greenhouse–Geisser adjustments of the *p* values are reported. We set the level of significance at *p* < 0.05. We performed statistical analyses in SPSS version 27.0 (IBM Corp. Armonk, NY, USA). The effect size was evaluated with eta partial squared (η_p_^2^), where 0.01 < η_p_^2^ < 0.06 constitutes a small effect, 0.06 < η_p_^2^ < 0.14 indicates a medium effect, and η_p_^2^ > 0.14 represents a large effect [17].

## 3. Results

The participants lifted on average 106 ± 25 kg at 1-RM, with a mean velocity of 0.23 ± 0.06 m/s. In all 1-RM attempts, there was a sticking region in the upward movement. After the sticking region, v_max2_ was higher than v_max1_ (Table 1). Time to v_max1_, v_min_, and v_max2_ and vertical distances at these points are presented in Table 1. The total lifting height was 0.486 ± 0.072 m.

**Figure 2:**
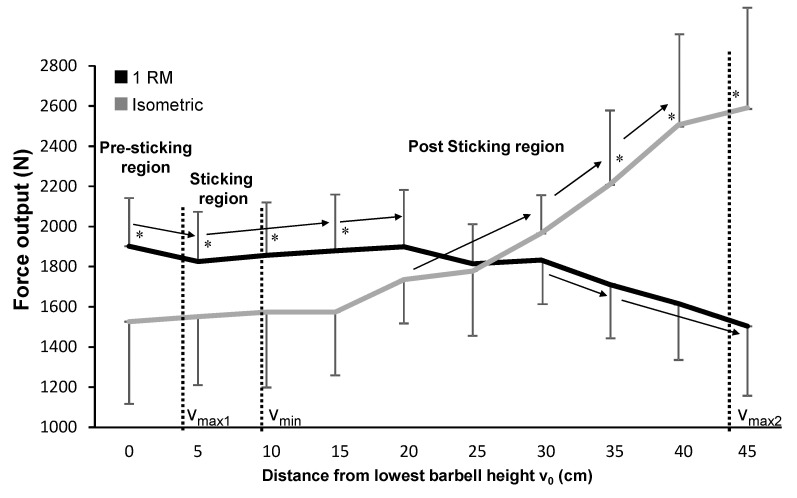
Mean (± standard deviation) maximal force output at the different vertical distances from the lowest barbell position for the one-repetition maximum (1-RM) squat attempt and the isometric contractions. * Indicates a significant difference (*p* < 0.05) in force output between the 1-RM and isometric attempts at this distance. → Indicates a significant difference (*p* < 0.05) in force output between these two distances for this condition.

A comparison of the force output between 1-RM and isometric squat attempts showed no significant effect between the two conditions (F = 0.01, *p* = 0.988, η_p_^2^ < 0.01). However, there was a significant height effect (F = 15.4, *p* < 0.01, η_p_^2^ = 0.66) and a significant condition × height interaction (F = 48.5, *p* < 0.001, η_p_^2^ < 0.86). Post hoc comparison showed the force output during the different isometric contraction heights were approximately the same over the first 20 cm and then increased with each height, while we observed a different development during 1-RM. Specifically, force output decreased from the lowest barbell point to 5 cm (from the pre-sticking to the sticking region), followed by a significant increase from 5 to 15 cm and 20 cm (from the sticking to the post-sticking region). Between 20 and 30 cm, force stayed relatively constant, after which force output decreased again. These different developments between the two conditions resulted in significantly higher force output in 1-RM for the first four heights, while in the last three heights the force output during the isometric contractions was higher (Figure 2).

**Figure 3:**
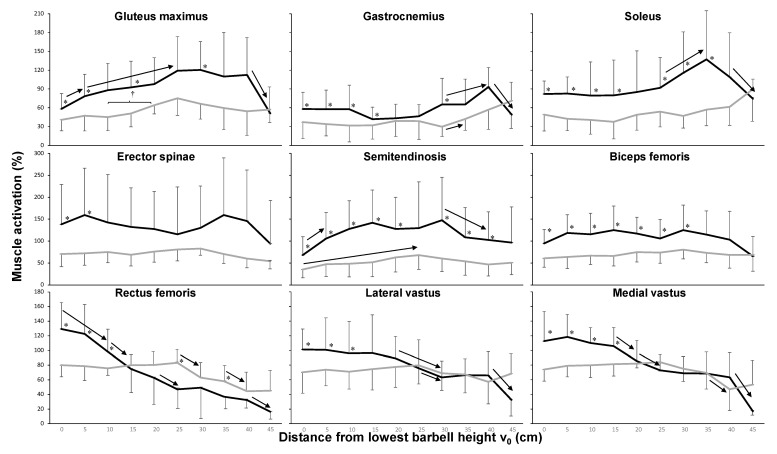
Mean (± standard deviation) muscle activities of the nine measured muscles at the different vertical distances from the lowest barbell position in the one-repetition maximum (1-RM) squat attempt and the isometric contractions. * Indicates a significant difference (*p* < 0.05) in muscle activity between the 1-RM and isometric attempts at this distance. → Indicates a significant difference (*p* < 0.05) in force output between these two distances for this condition.

The EMG-position profile between the two conditions showed a significant effect (F ≥ 5.3, *p* ≤ 0.05, η_p_^2^ ≥ 0.40) for all muscles except the rectus femoris (F = 0.19, *p* = 0.67, η_p_^2^ = 0.02). Height also had a significant effect on most muscles (F ≥ 2.4, *p* ≤ 0.024, η_p_^2^ ≥ 0.23), except the erector spinae (F = 0.34, *p* = 0.96, η_p_^2^ = 0.04) and the biceps femoris (F = 1.03, *p* = 0.42, η_p_^2^ = 0.11). In addition, there was a significant condition × height interaction for all three quadriceps muscles (F ≥ 2.2, *p* ≤ 0.040, η_p_^2^ ≥ 0.22). Post hoc comparison revealed that in most muscles, activity was higher during the 1-RM lift compared with the isometric contractions, especially at the 3–4 lowest heights (Figure 3). The gluteus maximus showed increased activity from the start to 25 cm, especially in the 1-RM condition, after which it decreased again for the last 5 cm of the 1-RM condition. The calf muscles (gastrocnemius and soleus) presented elevated activity from 25 to 30 cm and decreased activity in the last 5 cm of the 1-RM lift. For the quadriceps muscles, activity in the isometric contractions was stable until the 25 cm height, after which it decreased; during 1-RM, activity was higher at the beginning compared with the isometric contractions at those heights and decreased equally over heights for the rectus femoris. The vastus muscle showed decreased activity during 1-RM from 15 to 25 cm (medial vastus) and 20 to 30 cm (lateral vastus) and at the last height (Figure 3). The semitendinosus had lower activity at the deepest point of the lift and decreased again from 30 to 40 cm in the 1-RM condition (Figure 3).

**Figure 4:**
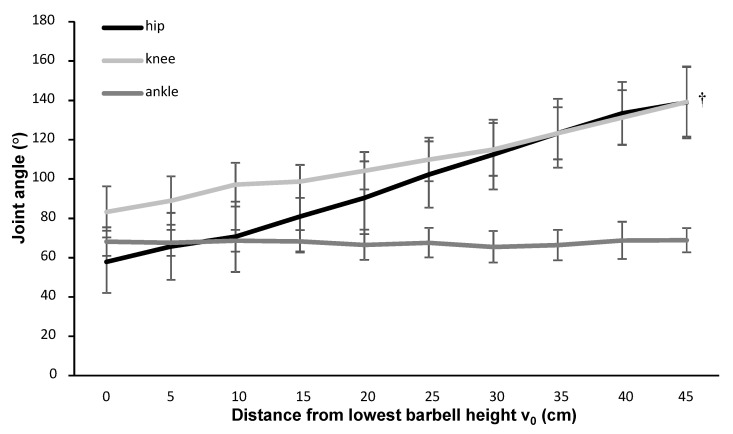
Mean (± standard deviation) joint angles of hip extension, knee extension, and ankle dorsal flexion at different vertical distances from the lowest barbell position in the isometric contractions. ^†^ Indicates a significant difference (*p* < 0.05) in joint angle between each adjacent height for knee and hip extension.

The joint angles increased significantly at each height of each isometric contraction for knee and hip extension (F ≥ 69.2, *p* ≤ 0.01, η_p_^2^ ≥ 0.91), but there was not a significant change in the ankle plantar flexion angle among the different heights (F = 0.81, *p* = 0.60, η_p_^2^ = 0.11, Figure 4).

## 4. Discussion

The purpose of this study was to investigate if the origin of the sticking region in squats is caused by a region of lower force output by comparing isometric squats at different heights with the regular maximal 1-RM squat. In both conditions, there was a region of lower force output during the sticking region (Figure 2); this finding supports the hypothesis that the sticking region is due to the position of mechanical disadvantage. However, the force output between the two conditions showed the opposite development at different heights. Furthermore, muscle activity was for most muscles higher during 1-RM than the maximal isometric contraction at different heights. The quadriceps muscle activity decreased, while the gluteus, semitendinosus, and calf muscle activity increased with increasing height.

The force output in both the 1-RM attempt and the isometric contraction condition showed that the force was lower in the sticking region (Figure 2). During the 1-RM attempt, the force decreased from the pre-sticking region to the sticking region and increased again at 15 cm, while in the isometric attempts force output over the first 20 cm (the pre-sticking, the sticking, and the first part of the post-sticking region) remained stable. Based on the maximal isometric force output at these heights, we can conclude that the occurrence of the sticking region in squats is the result of a weak position in this length-force characteristic of the musculoskeletal system, confirming the reasoning of [2] and van den Tillaar, Saeterbakken and Ettema [5], who observed this phenomenon in the bench press exercise. The force output decrease from 0 to 5 cm (from the pre-sticking to the sticking region) during 1-RM can be the result of potentiation [13] The major part of the enhanced force in the 1-RM condition disappeared after about 0.46 ± 021 s. This is around the same time that the influence of potentiation diminishes [13,18]. Thus, the force profiles indicate that diminishing potentiation occurs in the 1-RM squat; [5] showed the same outcome in the bench press exercise. The force output was higher over the first 20 cm in the 1-RM attempt compared with the isometric contractions at these heights. This outcome was probably the result of greater muscle activation of especially the quadriceps muscles at these heights (Figure 3), which also decreased with increasing height.

Of note, there was an increase in force output immediately after the sticking region in squats, although van den Tillaar et al. [5] did not observe this phenomenon in the case of a bench press exercise. This is explainable by the fact that in squats the second peak velocity (v_max2_) is in general much higher than the first peak velocity (v_max1_) due to the increased gluteus activity later in the lift [7]. In the bench press exercise, v_max2_ is the same or lower than v_max1_ because there are no other large muscles that take over during the lift [19]. The lower force output from 30 cm and above in the 1-RM condition compared with the isometric contractions may be the combined result of fatigue and shortening velocity, as well as the elimination of the requirement for a maximal effort. In the 1-RM attempt, the muscles contract concentrically over a long time after already being actively eccentric during the descending phase. We used the highest force output averaged over approximately 1 s in the isometric contractions for further analysis; this value is not influenced as much by fatigue as in the 1-RM attempt. Furthermore, after having succeeded the weakest point (sticking region), the weight merely needs to be pushed up, not at a maximal velocity. Thereby, the requirement of a maximal effort is no longer necessary, as shown by the decrease at the last height in 1-RM in most muscles, together with the purpose of decelerating the barbell at the highest point (Figure 3).

Most muscles had greater activation during the 1-RM condition compared with the isometric attempts (Figure 3), which is probably caused by greater activation and potentiation at the start of the ascending phase [13]. This phenomenon was especially visible in the quadriceps: the activity was greater, but subsequently decreased, over the first 4–5 barbell heights for the 1-RM condition, while it remained stable and lower at the similar barbell heights for the isometric condition. At these heights, gluteus activity increased with increasing height, a finding similar to van den Tillaar [7], who showed that gluteus maximus activity increased from the sticking to post-sticking region (Figure 3). Also, he showed that the quadriceps activation decreased from the pre-sticking–sticking region to the post-sticking region during 1-RM. In the isometric contraction condition, quadriceps activity was stable for the first 25 cm, which is comparable with the lifting distance at v_min_ (0.22 ± 0.07 m) in the squat study of van den Tillaar [7]. This finding indicates that the quadriceps muscles are the prime mover muscles during the first part of the ascending phase (pre-sticking and sticking phases). In the pre-sticking and sticking regions, only the vastus muscles of the quadriceps muscles contributed to positive work [20]. As Robertson, Wilson and Pierre [20] reported, the rectus femoris is a bi-articular muscle (knee extensor and hip flexor); it cannot produce much positive work because it acts eccentrically through the pre-sticking region, where most of the external work of the knee extensors was carried out. They showed that rectus femoris muscle lengths did not vary much (<2%) during the lift and that the rectus femoris may have acted to prevent unwarranted hip extension. A hip extension that is too early is undesirable because it could cause an anticlockwise rotation moment of the barbell and thereby increase the danger of slipping the barbell backward. Therefore, the gluteus muscles cannot be too active at the lowest heights (pre-sticking and sticking regions in 1-RM). Furthermore, at these heights, the hip angle is so low (60–70°), as shown in the isometric contractions (Figure 4), that the large gluteus muscle length gives a mechanical disadvantage such that the capacity to exert force was reduced [7,20]. This condition also gives the barbell weight position high up on the back a large moment arm around the hip joint; this condition has a negative influence (extra stretch) upon the gluteus maximus muscle activity and the erector spinae. Consequently, as van den Tillaar [7] showed, hip extension velocity is very low in this part of the upward movement, but it does reach maximal velocity around the v_max2_, which is also increased by gluteus activation during the 1-RM and isometric contractions at 25 cm (Figure 3). The same reasoning (long muscle length at lowest heights and creating a moment when activating too early) can be used for the calf muscles. Therefore, calf muscle activation in 1-RM only increased from 25 to 30 cm, which caused a plantar flexion that reached its maximal velocity at around v_max2_ [7].

The barbell kinematics (velocities and time) were comparable with the earlier studies on the sticking region in squats [7,9,10,12], thereby indicating that the measured 1-RM was the participants’ true 1-RM. However, the height at which the sticking region occurred (from 5 to 10 cm) was a bit short and only comparable with van den Tillaar, Andersen and Saeterbakken [12], who used as criteria only 90-degree knee flexion as the maximal depth and not the IPF rules. In the present study, most participants went a bit deeper (84.9 ± 11.8°), but not all due to the limitations of the Smith machine (no horizontal movements possible) that cause participants to adapt their range of motion during the lifts compared to what they were used to during free-weight barbell lifting. Thereby, only a short sticking region occurs. The difference in sticking region occurrence (distance) between the different studies can be explained by the joint angles. In the present study, the knee angles for the different events (Table 1) were comparable with van den Tillaar et al. [12], while the knee angles were lower in van den Tillaar [7] at v_0_ and v_max1_. However, when comparing at v_min_ and v_max2_, the present study and the two previous studies [7,12] found comparable knee angles (v_min_: 100–103°, v_max2_: 136–140°) and ankle angles (v_min_: 71–74°, v_max2_: 78–79°). In other words, these events occur at similar joint angles. The discrepancy in hip angles with van den Tillaar [7] could be due to the use of the Smith machine, which limits the barbell to move only vertically—no horizontal movements are possible. By limiting the degrees of freedom, it is possible that some muscle can be activated more than they could with free-weight squat lifting. For example, in the Smith machine the gluteus and calf muscles can be activated without causing a rotation moment on the barbell and thereby lead to a smaller sticking region. Thus, the hip flexion angles could be different during the lift due to this limitation of the degrees of freedom.

During the isometric contractions, hip and knee extension angles increased with each increasing height, while no major changes occurred in the ankle angle, which is not in line with the 1-RM pattern in which dorsiflexion increases with height (Table 1). This phenomenon also explains why calf muscle activity is higher at 25–35 cm in 1-RM compared with the isometric contraction (Figure 3). Although the stance position was controlled at each attempt, due to the use of a Smith machine, adaptations during the isometric contractions were mainly done in the knee and hip joint and not in the ankle joint. Thereby, the calf muscle activity levels at the greatest heights between 1-RM and the isometric contractions are perhaps not good to compare with each other due to the differences in ankle joint angles at these heights (around v_max2_). This is a limitation of the study, and it must be controlled in future studies.

Another limitation is that we did not conduct inverse dynamics due to insufficient equipment. Performing inverse dynamics could give more information about the moments and forces around the different joints; this information could explain the muscle activation at these heights. Therefore, in future studies, 1-RM free-weight squats and isometric contractions at different heights, while controlling joint angles and including inverse dynamics analyses, should be conducted to provide a better view of the reasons why the sticking region occurs when performing squats.

## 5. Conclusions

Force output decreased at the barbell heights representing the sticking region for the 1-RM trial, indicating that the sticking region is a poor biomechanical force region. Our findings suggest that the sticking region is a poor biomechanical force region due to the potentiation of the vastus muscles and the co-contraction between the hip and knee extensors together with the plantar flexors during Smith machine squats. The activity of the quadriceps muscles decreased and the activity of the gluteus maximus increased during the sticking region. Information from the present study could help coaches and athletes understand what causes the sticking region and potentially how to enhance squat performance during 1-RM lifts.

## Figures and Tables

**Table 1:** Mean (±standard deviation) barbell kinematics and joint angles at the different events of the sticking region during the one-repetition maximum average for all participants

Event	v_0_	v_max1_	v_min_	v_max2_
Barbell height (m)	0	0.046 ± 0.022	0.095 ± 0.047	0.433 ± 0.086
Barbell velocity (m/s)	0	0.17 ± 0.07	0.13 ± 0.07	0.59 ± 0.14
Time (s)	0	0.46 ± 0.21	0.84 ± 0.32	2.07 ± 0.81
Hip extension (°)	61.0 ± 20.7	69.6 ± 24.6	76.9 ± 27.2	129.6 ± 22.5
Knee extension (°)	84.9 ± 11.8	93.3 ± 11.2	100.0 ± 9.4	140.5 ± 8.1
Ankle plantar flexion (°)	67.0 ± 8.9	71.1 ± 8.0	73.7 ± 5.6	78.4 ± 5.3

## Data Availability

The raw data supporting the conclusions of this article will be made available by the authors, without undue reservation.
